# A new bioinformatic insight into the associated proteins in psychiatric disorders

**DOI:** 10.1186/s40064-016-3655-6

**Published:** 2016-11-14

**Authors:** Wenlong Zhao, Wenjing Yang, Shuanglin Zheng, Qiong Hu, Ping Qiu, Xinghua Huang, Xiaoqian Hong, Fenghua Lan

**Affiliations:** 1 Department of Neurology, Affiliated Dongfang Hospital of Xiamen University (Fuzhou General Hospital), Fuzhou, Fujian People’s Republic of China; 2 Department of Clinical Genetics and Experimental Medicine, Fuzhou General Hospital, No. 156, Xier Huan Road, Gulou District, Fuzhou, 350025 Fujian People’s Republic of China

**Keywords:** Psychiatric diseases, Bioinformatics, Molecular biomarkers, Therapy, Signaling pathways

## Abstract

**Background:**

Psychiatric diseases severely affect the quality of patients’ lives and bring huge economic pressure to their families. Also, the great phenotypic variability among these patients makes it difficult to investigate the pathogenesis. Nowadays, bioinformatics is hopeful to be used as an effective tool for the diagnosis of psychiatric disorders, which can identify sensitive biomarkers and explore associated signaling pathways.

**Methods:**

In this study, we performed an integrated bioinformatic analysis on 1945 mental-associated proteins including 91 secreted proteins and 593 membrane proteins, which were screened from the Universal Protein Resource (Uniport) database. Then the function and pathway enrichment analyses, ontological classification, and constructed PPI network were executed.

**Results:**

Our present study revealed that the majority of mental proteins were closely related to metabolic processes and cellular processes. We also identified some significant molecular biomarkers in the progression of mental disorders, such as HRAS, ALS2, SLC6A1, SLC39A12, SIL1, IDUA, NEPH2 and XPO1. Furthermore, it was found that hub proteins, such as COMT, POMC, NPS and BDNF, might be the potential targets for mental disorders therapy. Finally, we demonstrated that psychiatric disorders may share the same signaling pathways with cancers, involving ESR1, BCL2 and MAPK3.

**Conclusion:**

Our data are expected to contribute to explaining the possible mechanisms of psychiatric diseases and providing a useful reference for the diagnosis and therapy of them.

## Background

Psychiatric disorders are generally regarded as neuropsychological and neurobehavioral lesions, and the impaired ability to understand new or complicated information. It is estimated that more than 450 million people worldwide suffer from various mental disorders, among which major depression will be the most debilitating one by 2030 (World Health Organization 2010). Also, the appearance of psychiatric disorders tends to take place in childhood with increasing prevalence and incidence. In addition, males with intellectual disability were even found to have an elevated risk of leukemia, brain, stomach, corpus uteri and colorectal cancers (Sullivan et al. 2004). However, the etiopathogenesis of many psychiatric diseases is still unclear.

Two thirds of psychiatric patients take physical examination at primary care facilities. They always complain about the lack of energy as well as general aches and pains, so that their doctors usually focus on the organic disorders but neglect the mental problems. Moreover, most kinds of psychiatric disorders often present similar symptoms, which frequently delays the early evaluation of neurodevelopment status and disease progression. To this day, the diagnosis of psychiatric diseases has mainly depended on the daily living experience of patients’ families and the subjective expression of patients. There seems not to be available biological tests objectively assessing the association between clinical symptoms and underlying molecular mechanisms.

Recently, with the development of bioinformatic technologies, we can get further insights into the pathogenesis of psychiatric disorders from the perspective of cells, circuits, and pathophysiological processes. And the recent findings of proteomic biomarker tests have made it more reliable and effective for the diagnosis and treatment of psychiatric disorders. For example, C-reactive protein (CRP) has been used as a biomarker to predict depression, and the increased CRP expression level was identified as an independent risk factor for de novo depression in women (Lopresti et al. 2014; Wium-Andersen et al. 2013). Besides, cytokines, neopterin, malondialdehyde, isoprostanes, and serum S100B have also been reported to be reliable and sensitive biomarkers of mood disorder. (Howgren et al. 2009; Rybka et al. 2013; Maes et al. 2012; Chung et al. 2013; Milaneschi et al. 2013; Schroeter et al. 2013). Because multiple biological process overlap in the progression of psychiatric diseases, greater successes may be made on mental disease treatment by targeting the early biomarkers and the crucial signaling pathways. These successes should also be useful for exploring underlying mechanisms of mental disorders.

In this study, we aimed to explore the potential proteomic biomarkers and pathological mechanisms in psychiatric disorders with the help of bioinformatic tools. First, we performed a comprehensive bioinformatic analysis on the associated proteins from UniProt database to determine their significant characteristics. Then the functional enrichment analyses and constructed PPI networks were carried out to identify key genes, proteins and signaling pathways. Psychiatric disorders investigated in this study were referred to autism spectrum disorder (ASD), schizophrenia (SCZ), bipolar disorders, major depression, anxiety, phobia, affective disorder, obsessive compulsive disorder, personality disorder and mental retardation psychiatry. Proteins with altered expression which influence the physiological and pathological progression of mental disorders may be involved in certain pathways.,. And once these pathways are made clear, it will help us know better about the pathogenic mechanisms and develop the targeted therapeutic strategies. Therefore, bioinformatic classifications should be utilized as useful references for identification and characterization of biomarkers, which will be expected to give a new direction to psychiatry research.

## Results

### Psychiatric-disease-associated proteins were screened and classified

We screened reviewed proteins from the UniProt database and collected 2317 proteins associated with mental disorders. The retrieved proteins from various psychiatric disorders were classified as follows: mental retardation (585 proteins), intellectual disability (425 proteins), cognitive disorder (94 proteins), autism (437 proteins), schizophrenia (413 proteins), bipolar disorder (219 proteins), depression (63 proteins), anxiety (8 proteins), phobia (51 proteins), obsessive compulsive disorder (7 proteins) and personality disorder (2 proteins). Because there are 372 proteins counted repeatedly among them, 1945 proteins associated with human psychiatric diseases were obtained finally.

### The associated proteins and signaling pathaways were identified by bioinformatic analysis

**Fig. 1:**
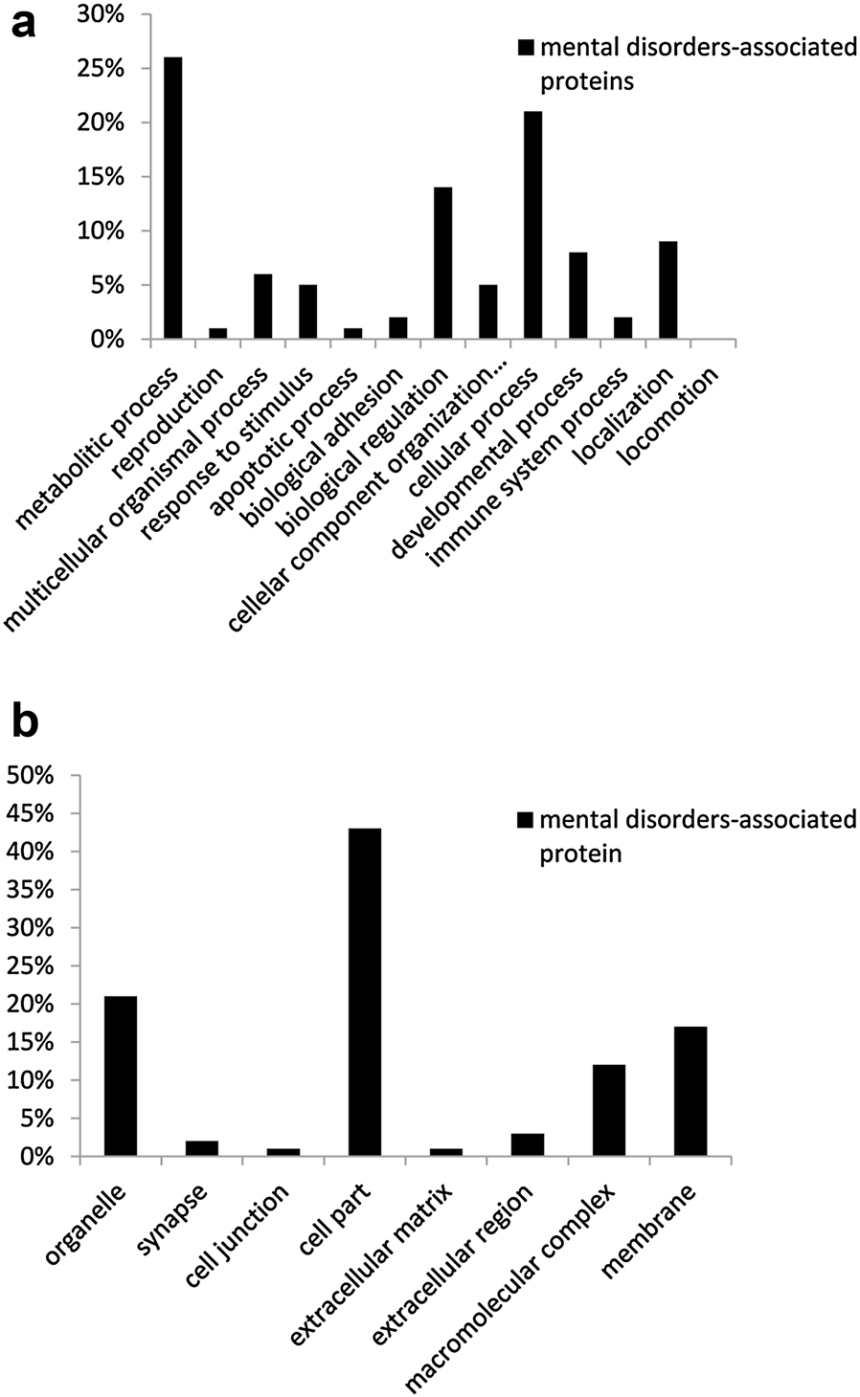
Broad functional classification of selected mental disorders-associated proteins. **a** Biological processes, **b** cellular component

After the DAVID functional annotation clustering, a total of 1075 GO terms were found to be associated with psychiatric diseases proteins. The implicative pathways of GO (top 20) were shown in Table 1. The result demonstrated that mental-disorder-associated proteins mainly enriched in 2 GO categories including biological process (BP) and cellular component (CC). In the BP group, the majority (26%) of the proteins were concerned with metabolic processes, followed by cellular processes (21%). While the proteins in the CC group were comprised of cell part (43%), organelle (21%) and membrane (17%) (Fig. 1). And the top 5 pathways significantly involved in GO terms contained transmission of nerve impulse, neuron projection, synaptic transmission, learning or memory and behavior.

**Table 1:** Top 20 GO function enrichment analysis of psychiatric diseases associated protein

Category	Term	Count	P value
Biological process	GO:0019226 : transmission of nerve impulse	91	2.89E−32
Cellular component	GO:0043005: neuron projection	88	8.81E−32
Biological process	GO:0007268: synaptic transmission	81	3.90E−30
Biological process	GO:0007611: learning or memory	50	6.30E−30
Biological process	GO:0007610: behavior	102	2.91E−29
Cellular component	GO:0030424: axon	52	5.71E−24
Cellular component	GO:0042995: cell projection	114	9.57E−23
Biological process	GO:0007267: cell–cell signaling	102	1.18E−20
Biological process	GO:0044057: regulation of system process	68	7.66E−20
Biological process	GO:0007612: learning	30	1.37E−19
Cellular component	GO:0045202: synapse	72	1.38E−19
Biological process	GO:0031644: regulation of neurological system process	46	3.97E−19
Biological process	GO:0042596: fear response	18	4.61E−19
Biological process	GO:0051969: regulation of transmission of nerve impulse	44	3.08E−18
Biological process	GO:0050877: neurological system process	153	6.80E−18
Biological process	GO:0030900: forebrain development	44	1.22E−17
Cellular component	GO:0030425: dendrite	45	1.25E−17
Biological process	GO:0050804: regulation of synaptic transmission	41	3.77E−17
Biological process	GO:0033555: multicellular organismal response to stress	23	4.65E−17
Biological process	GO:0032990: cell part morphogenesis	57	5.83E−17

By performing the KEGG analysis for enriched proteins, we obtained 30 significant KEGG pathways with P value <0.05, and we showed the top 20 in Table 2. The top 5 significant pathways were listed as follows: Neuroactive ligand-receptor interaction, Calcium signaling pathway, Long-term potentiation, Long-term depression, Prostate cancer.

**Table 2:** The enriched KEGG pathways of psychiatric disorders

Term	Description	Count	P value
KEGG:04080	Neuroactive ligand-receptor interaction	54	2.15E−10
KEGG:04020	Calcium signaling pathway	34	6.88E−06
KEGG:04720	Long-term potentiation	18	3.01E−05
KEGG:04730	Long-term depression	18	3.70E−05
KEGG:05215	Prostate cancer	19	3.37E−04
KEGG:04540	Gap junction	18	9.74E−04
KEGG:04150	mTOR signaling pathway	12	0.003294
KEGG:05012	Parkinson’s disease	21	0.004578
KEGG:05216	Thyroid cancer	8	0.008593
KEGG:05220	Chronic myeloid leukemia	14	0.008781
KEGG:00250	Alanine, aspartate and glutamate metabolism	8	0.012514
KEGG:04012	ErbB signaling pathway	15	0.012769
KEGG:05218	Melanoma	13	0.014087
KEGG:05214	Glioma	12	0.014554
KEGG:05200	Pathways in cancer	40	0.015047
KEGG:00563	Glycosylphosphatidylinositol(GPI)-anchor biosynthesis	7	0.015127
KEGG:05010	Alzheimer’s disease	23	0.016819
KEGG:04916	Melanogenesis	16	0.017224
KEGG:04142	Lysosome	18	0.017275

Analysis of tissue specific proteins revealed that most of the proteins were expressed in brain (49%), epithelium (15.6%) and placenta (17%). In addition, there are 4 proteins members of the VCX family expressed exclusively in testis.

### Secretory proteins and cell surface proteins were identified

After retrieval of the “LOCATE” database, membrane organization analysis indicated that 1261 proteins were soluble proteins including 91 secreted proteins, and that 593 proteins were membrane proteins containing 76 type I proeins, 168 type II proteins and 349 multiple transmembrane protein.

### Hub proteins were identified by PPI network

**Fig. 2:**
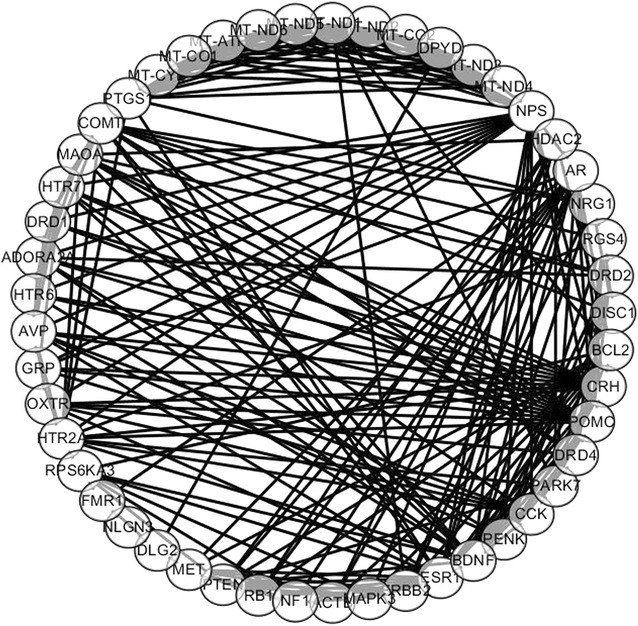
The constructed PPI network of psychiatric diseases. Nodes represent proteins, edges represent interactions between two proteins

PPI networks were established by Cytoscape software including 329 nodes and 1194 edges based on STRING database. Then, the degree of all nodes was calculated and the proteins whose node degree >20 were shown as follows: POMC (proopiomelanocortin, Degree = 41), NPS (neuropeptide S = 38), ESR1 (estrogen receptor 1, Degree = 35), BDNF (brain-derived neurotrophic factor = 32), BCL2 (B-cell CLL/lymphoma 2 = 31), PTEN (phosphatase and tensin homolog, Degree = 29), AR (androgen receptor = 29), MAPK3 (mitogen-activated protein kinase 3, Degree = 27), HDAC2 (histone deacetylase 2 = 27), AVP (arginine vasopressin = 24), CRH (corticotropin releasing hormone = 24), COMT (catechol-O-methyltransferase, Degree = 22), HTR2A (5-hydroxytryptamine receptor 2A = 21), ACTB (actin, beta = 21), DPYD (dihydropyrimidine dehydrogenase = 21), MT-ND1 (mitochondrially encoded NADH dehydrogenase 1 = 21), DRD2 (dopamine receptor D2 = 21), CCK (cholecystokinin = 20), HTR7 (5-hydroxytryptamine receptor 7 = 20). These hub proteins were displayed in Table 3 and the associated PPI networks were shown in Fig. 2.

**Table 3:** Degree of each hub node in the PPI network

Gene symbol	Degree
POMC	41
NPS	38
ESR1	35
BDNF	32
BCL2	31
PTEN, AR	29
HDAC2, MAPK3	27
AVP, CRH	24
COMT	22
HTR2A, ACTB, DPYD, MT-ND1, DRD2	21
CCK, HTR7	20

## Discussion

The diagnosis of psychiatric disorders always lack biological gold standards because of the complicated nosology. The huge phenotypic variability in population also bring great challenges to explore credible biomarkers in the fields of drug abuse and neuropsychiatry researches. However, with the advancement of bioinformatic technology, a plenty variety of proteins have been explored to define different progression stages of mental diseases. We made full use of the information of psychiatric-disease-associated proteins from the public databases and performed comprehensive bioinformatic analyses to determine sensitive biomarkers and significant signaling pathways. Our work is expected to provid new insights into the diagnosis of psychiatric diseases and investigate the underlying mechanisms. In future, more bioinformatic data of mental diseases should be needed to evaluate risks and benefits of specific therapeutic approaches and to enhance drug research and development.

In our study, 1945 mental-associated proteins were screened from the public database. Then we performed an integrated bioinformatic analysis to determine the biological relationship between these proteins and psychiatric diseases. Ontological analysis demonstrated that most of the proteins, which could be classified into various functional groups, may participate in different biological activities of brain. Functional clustering analysis also implied that these mental-associated proteins were significantly related to five GO terms: transmission of nerve impulse, neuron projection, synaptic transmission, learning or memory and behavior. We found transmission of nerve impulse was the most important term enriched in psychiatric diseases, which played a fundamental role in dendritic morphology and electrophysiological properties. And the second most important term neuron projection is associated with the transcription of ribosome-bound mRNA (Cook-Snyder et al. 2015). More than 80 proteins were identified in neuron projection term, and these proteins may influence metabolic process or cellular process in mental disorders, such as STIL (SCL/TAL1 interrupting locus) and XPO1 (exportin 1). Besides, ALS2 (alsin Rho guanine nucleotide exchange factor) and SLC6A1 (solute carrier family 6 member 1), which were the most prominent proteins related to the majorty of top GO terms, could be valuable for the diagnosis and targeted therapy of psychiatry,. Many studies reported that common motor neuron diseases have a close relationship with mutations in ALS2, resulting in juvenile lateral sclerosis and infantile-onset ascending spastic paralysis (Sheerin et al. 2014; Eker et al. 2014). The neurotransmitter r-aminobutyric acid (GABA) transporter 1, encoded by SLC6A1 gene, was verified as the most prominent biomarker in neural signal transduction pathways (Berg and Geschwind 2012). Nicola reported that even a tiny deletion of SLC6A1 gene could result in intellectual disability and multiple congenital anomalies (Dikow et al. 2014).

Through analysis of KEGG pathway enrichment, we finally got 30 significant pathways. Most of these pathways can be classified into two categories: neural signal transduction and cancer pathways. The neural signal transduction contains neuroactive ligand-receptor interaction pathway, calcium signaling pathway, long-term potentiation (LTP) and depression (LTD), gap junction, lysosome, Alanine, aspaetate and glutamate metabolism. The most important neuroactive ligand-receptor interaction pathway has been applied into the assessment of antipsychotic treatment and the analysis of neuropsychiatric disorder model (Adkins et al. 2012; Kong et al. 2015), which is enriched by TSPO, THRA, THRB, GABRB3, GRIK1, GABRB2, GRIK2, TRPV1, GABRB1, HTR6, OXTR. HRAS (harvey rat sarcoma viral oncogene homolog) was a predominant protein participating in 18 significant pathways. It may play key roles in psychiatric disorders with pathological alteration. Schwartz mentioned that HRAS functioned in synaptic long-term potentiation (LTP) and memory formation (Schwartz et al. 2013). It was demonstrated that the regulation of long-term potentiation is important for adult neurogenesis, synaptic plasticity, and learning and memory (Madroñal et al. 2010; Cooke and Bear 2010; Park et al. 2015; Jing et al. 2015). Therefore, HRAS is an ideal marker for psychiatric disorders diagnosis. While cancer pathways contained prostate cancer, thyroid cancer, chronic myeloid leukemia, melanoma, glioma, ErbB signaling pathway, and mTOR signaling pathway. These pathways remind us a fire-new angle to explore useful information from a mass of known proteins which were ignored before. MAPK3 is known as extracellular signal-regulated kinases (ERKs) and almost appears in all pathways. This gene always regulates a variety of cellular processes such as cell cycle progression, differentiation and proliferation. The overexpression of MAPK3 may indicate the pathogenesis of fear, anxiety and related psychopathological conditions.

After the membrane organization analysis, 91 secreted proteins were predicted, which could be candidate proteins for diagnosis of heritable mental diseases. For example, SLC39A12 (solute carrier family 39, member 12), a putative zinc transporter gene, is highly expressed in adult brains. It can encode ZIP12 (zinc transporter 12) protein which is required for neurulation and neuronal development (Chowanadisai et al. 2013; Chowanadisai 2014). Any other protein including SIL1 (nucleotide exchange factor) precursor protein, IDUA (iduronidase), APP (amyloid beta precursor protein), NEPH2 (kin of IRRE-like protein), AOF2 (amine oxidoreductase), and WDR81 (WD repeat domain 81) proteins are expected to be diagnostic markers or therapeutic targets. And we found that 593 membrane proteins were transporters, receptors, enzymes and adhesion molecules, which were involved in nerve cell interactions, signaling transduction and energy metabolism.

Hub nodes have more complicated interactions with mental diseases compared with other proteins, implying that they paly a crucial part in mental disorders (Langfelder and Mischel 2013). Therefore, identification of the hub proteins may enhance the assessment of disorders progression, neurodevelopment status, and therapeutic approaches. The following genes: POMC, NPS, ESR1, BDNF, BCL2, PTEN, AR, MAPK3, HDAC2, AVP, CRH, COMT, HTR2A, ACTB, DPYD, MT-ND1, DRD2, CCK, HTR7 were discovered as the genes that encode hub proteins in the PPI network. The POMC gene had a highest degree of 41, encoding the adrenocorticotropin hormone (ACTH) peptide, which brought our attention to major depressive disorders and the regulation of hypothalamic–pituitary–adrenal (HPA) axis function. POMC gene could be a valuable marker for assessing the response to antidepressant treatment (Chang et al. 2015). Furthermore, the polymorphisms of COMT gene are associated with sporadic schizophrenia and mood disorder, respectively (Li et al. 2015; Pandolfo et al. 2015). COMT inhibitors had been used to ameliorate symptoms of parkinson’s disease (Müller 2015). Therefore, POMC and COMT are both potential pharmacological targets for treating psychiatric disorders.

The NPS and BDNF genes also have multidirectional physiological activities in brain. Recent studies showed that NPS was associated with the regulation of food intake, fear, addiction and anxiety (Oishi et al. 2014; Slattery et al. 2015; Wegener et al. 2012). Dine et al. showed NPS application to mouse brain slices activated neurotransmission and plasticity in hippocampal CA3 and CA1 synapses (Dine et al. 2013). And the strong anxiolytic effect of NPS may be concerned with the increased dopamine releasing in medial prefrontal cortex (Lukas and Neumannid 2012; Si et al. 2010). The BDNF gene encodes a few proteins of nerve growth factor families, involving the process of neurogenesis and the realization of neuroprotective functions. It was also found that BDNF played a key role in memory and learning organization and motor function. The expression of BDNF protein always decreases in patients with degenerative and vascular dementias, anxiety, affective and behavioral disorders (Levada and Cherednichenko 2015). Jehn et al. indicated that the low-level expression of BDNF was associated with cognitive impairment and short-term memory, coupled with the increased IL-6, which would be implicated in pathophysiology of depression patients (Jehn et al. 2015). NPS and BDNF could be used as neurobiological markers in pathological process, and the increased concentration may provide a potential novel treatment option.

Increased references in PubMed also provided us other hub proteins involved in cancer pathways, such as ESR1, PTEN, BCL2, AR and MAPK3. The down-regulation of BDNF-Akt-Bcl2 anti-apoptotic signaling pathway could be responsible for pathogenesis of autism. (Sheikh et al. 2010) Borges also reported that ERK1/2 signaling pathway effectively regulate depression, anxiety and emotion (Borges et al. 2015). Therefore, these potential targets involved in cancers may share the same signaling pathway with mental disorders. To ascertain the accurate roles of these genes may clarify pathological mechanisms and provide valuable information for psychiatric therapy.

## Conclusions

This study analyzed mental-associated proteins out of the reviewed database to explore the molecular mechanisms of psychiatric diseases by using bioinformatic methods. Our findings are expected to contribute to determining mental biomarkers, such as secreted proteins SLC39A12, SIL1, IDUA, APP, NEPH2, AOF2, and WDR81. And hub proteins, such as POMC, COMT, NPS, BDNF, also have the potential to be applied as targets for the diagnosis and treatment of mental disorders.

## Methods

### Screen of psychiatric diseases associated proteins

The UniProt (Release 2011_10, http://www.uniprot.org) was used to screen psychiatric diseases associated proteins. All human proteins were extracted from the UniProt database involving all annotations, such as “mental retardation”, “intellectual disability”, “autism”, “Schizophrenia”, “bipolar disorder”, “major depression”, “anxiety”, “phobia”, “affective disorder”, “obsessive compulsive disorder”, and “personality disorder”. Then the associated proteins were further filtrated manually from the downloaded data.

### Enrichment bioinformatic analysis

Protein IDs were submitted to DAVID (http://david.abcc.ncifcrf.gv/), a platform for functional analysis, such as gene ontologies, protein domains, and pathways. Of these, the enrichment analysis of GO categories includes biological process (BP), molecular function (MF), and cellular component (CC). The P value was computed by right-sided hypergeometric tests, GO terms were considered significance with P value <0.01, and KEGG pathways with P value <0.05. Protein IDs extracted from UniProt also were uploaded to PANTHER (www.pantherdb.org) to identify molecular function and protein class term through gene expression tools.

### Analysis of the membrane organization

The LOCATE (http://locate.imb.uq.edu.au/), a curated database, was used to describe membrane organization of proteins. Uniprot IDs of mental illness proteins were uploaded to the LOCATE server to select secretory and cell surface proteins, which are promising biomarkers.

### PPI network construction and hub proteins identification

The STRING database, a Search Tool to pre-compute global resource and evaluate protein–protein interactions information (von Mering et al. 2003), was used to construct PPI networks. And we primarily analyzed screened proteins of human psychiatric diseases by the UniProt database. Then, we performed analysis for PPI networks using Geniscape of Cytoscape software (version: 3.2.1) (Shannon et al. 2003). From a previous document on biological networks, numbers of PPI networks subjected to the scale-free attribution (Lamb et al. 2006). Therefore, connectivity degree was calculated by statistics and important nodes in PPI networks were obtained, namely hub proteins. Finally, the node degree >20 was selected as the threshold to obtain hub proteins in our study.

## Abbreviations

CRP: C-reactive protein
ASD: autism spectrum disorder
SCZ: Schizophrenia
BP: biological process
MF: molecular function
CC: cellular component
POMC: proopiomelanocortin
NPS: neuropeptide S
ESR1: estrogen receptor 1
BDNF: brain-derived neurotrophic factor
BCL2: B cell CLL/lymphoma 2
PTEN: phosphatase and tensin homolog
AR: androgen receptor
MAPK3: mitogen-activated protein kinase 3
HDAC2: histone deacetylase 2
AVP: arginine vasopressin
CRH: corticotropin releasing hormone
COMT: catechol-*O*-methyltransferase
HTR2A: 5-hydroxytryptamine receptor 2A
ACTB: actin, beta
DPYD: dihydropyrimidine dehydrogenase
MT-ND1: mitochondrially encoded NADH dehydrogenase 1
DRD2: dopamine receptor D2
CCK: cholecystokinin
HTR7: 5-hydroxytryptamine receptor 7
STIL: SCL/TAL1 interrupting locus
XPO1: exportin 1
ALS2: alsin Rho guanine nucleotide exchange factor
SLC6A1: solute carrier family 6 member 1
SLC39A12: solute carrier family 39, member 12
ZIP12: zinc transporter 12
SIL1: nucleotide exchange factor
IDUA: iduronidase
APP: amyloid beta precursor protein
NEPH2L: kind of IRRE-like protein
AOF2: amine oxidoreductase
WDR81: WD repeat domain 81
